# When the Good Syndrome Goes Bad: A Systematic Literature Review

**DOI:** 10.3389/fimmu.2021.679556

**Published:** 2021-05-25

**Authors:** Yiyun Shi, Chen Wang

**Affiliations:** ^1^ Department of Medicine, The Warren Alpert Medical School of Brown University, Providence, RI, United States; ^2^ Department of Internal Medicine, Morsani College of Medicine, University of South Florida, Tampa, FL, United States

**Keywords:** Good syndrome, immunodeficiency, prognosis, clinical subgroups, infections

## Abstract

**Background:**

Good syndrome is a rare adult-onset immunodeficiency characterized by thymoma and hypogammaglobulinemia. Its clinical manifestations are highly heterogeneous, ranging from various infections to autoimmunity.

**Objective:**

This study was to summarize patient characteristics, identify prognostic factors and define clinical subgroups of Good syndrome.

**Methods:**

A systematic literature review was conducted to include patients with Good syndrome identified in PubMed, Embase and Cochrane databases between January 2010 and November 2020. Logistic and Cox regressions were used to identify prognostic factors impacting outcomes. Clinical subgroups were defined by multiple correspondence analysis and unsupervised hierarchical clustering. A decision tree was constructed to characterize the subgroup placement of cases.

**Results:**

Of 162 patients included in the current study, the median age at diagnosis was 58 years and 51% were male. Type AB was the most common histological subtype of thymoma, and infections as well as concurrent autoimmune disorders were identified in 92.6% and 51.2% patients, respectively. Laboratory workup showed typical findings of combined immunodeficiency. Thymoma status (odds ratio [OR] 4.157, confidence interval [CI] 1.219-14.177, *p* = 0.023), infections related to cellular immunity defects (OR 3.324, 95% CI 1.100-10.046, *p* = 0.033), infections of sinopulmonary tract (OR 14.351, 95% CI 2.525-81.576, *p* = 0.003), central nerve system (OR 6.403, 95% CI 1.205-34.027, *p* = 0.029) as well as bloodstream (OR 6.917, 95% CI 1.519-31.505, *p* = 0.012) were independent prognostic factors. The 10-year overall survival was 53.7%. Cluster analysis revealed three clinical subgroups with distinct characteristics and prognosis (cluster 1, infections related to cellular immunity defects; cluster 2, infections related to other immunity defects; cluster 3, infections related to humoral and phagocytic immunity defects). A decision tree using infection types (related to humoral and cellular immunity defects) could place patients into corresponding clusters with an overall correct prediction of 72.2%.

**Conclusions:**

Infection type and site were the main prognostic factors impacting survival of patients with Good syndrome. We identified three subgroups within Good syndrome associated with distinct clinical features, which may facilitate the study of underlying pathogenesis as well as development of targeted therapy.

## Introduction

Good syndrome is an adult-onset acquired immunodeficiency, characterized by thymoma and hypogammaglobulinemia. Its underlying pathogenesis remains elusive. Patients always have low to absent peripheral B cells and impaired T-cell mediated immunity. Its clinical constellations are highly heterogeneous, ranging from various infections to concurrent autoimmune disorders. Recognition of the disease across a range of manifestations is challenging, commonly leading to diagnostic delay. Treatments are mainly supportive with antimicrobials and immunoglobulin replacement. Prognosis is believed to be worse compared with other adult immunodeficiencies (1, 2).

Data on Good syndrome are scarce due to its rarity. Most studies are case reports as well as small series, and the last systematic review was published in 2010 focusing on descriptions of clinical features (2). Therefore, the current systematic literature review aimed to summarize the clinical features, concurrent disorders, treatments, and outcomes of Good syndrome cases published since 2010. Independent prognostic factors impacting survival were explored. Given its spectrum of manifestations, we also sought to define disease subgroups sharing similar clinical features and prognosis, which may enable earlier diagnosis and more specific treatment.

## Methods

### Study Design, Search Strategy and Selection Criteria

Records were identified by searching PubMed, Embase and Cochrane databases between January 2010 and November 2020, with the terms “Good syndrome”, “Good’s syndrome”, “thymoma” AND “hypogammaglobulinemia”, “thymoma” AND “immunodeficiency”, as well as “thymoma” AND “infection”. We chose 2010 as the initial year because a previous systematic review already summarized cases published between 1956 and 2009 (2). In addition, cases reported in the recent decade were described in a more standardized manner, allowing better synthesis of the data. We further reviewed the reference lists of retrieved records to identify additional cases. Duplicated records were excluded first. Two authors (YS and CW) screened the title and abstract of each record independently, and full texts of the record deemed relevant were reviewed by both authors to reach a consensus for inclusion or exclusion. As there is no formal diagnostic consensus for Good syndrome, we used the presence of both thymoma and hypogammaglobulinemia as the minimal criteria to define Good syndrome, consistent with the previous systematic review (2). Cases with individual patient data were included. Exclusion criteria were an inappropriate type of record (review, conference abstract or non-English publication), an alternative diagnosis or a study of Good syndrome with aggregated data. Aggregated data of the Good syndrome cohort were summarized and reported separately (**Supplementary Table 1**) to allow comparison (3–6).

Primary aims of the current systematic review were to record the baseline clinical features (especially infection histories and related pathogens (**Supplementary Table 2**), laboratory findings, concurrent diseases, treatments, and mortality of patients with Good syndrome. Given the diversity of pathogens reported in these patients, they were grouped according to the major type of host immunity defect predisposing to the infection, including humoral, cellular, phagocytic and others (including pathogens not associated with a particular type of immunity defects or pathogen was not reported for the infection) (7–11). Details of pathogens in each category were shown in **Supplementary Table 3**. More advanced immunological workups, including vaccination response, lymphocyte proliferation to mitogens and respiratory burst test were not available in most cases. Prognostic factors impacting outcomes (mortality and survival) were explored. Clinical findings were also used to identify disease subgroups with similar manifestations and outcomes.

### Statistical Analysis

Data analysis was performed with SPSS version 26.0 (Armonk, NY, USA) and XLSTAT version 2020.1 version (New York, NY, USA). Continuous variables were summarized with median and interquartile range (IQR). Categorical variables were reported as numbers and percentages. Continuous variables were compared using Mann-Whitney test or Kruskal-Wallis test, and categorical variables were compared using Fisher’s exact test or chi-square test, as appropriate.

Binary logistic regression was used to identify prognostic factors impacting mortality for all patients (*n* = 162), and variables with both clinical and statistical relevance (*p* < 0.10) in univariate analysis were included in the multivariate model. For patients with clearly documented follow up duration (*n* = 109), overall survival was measured from the time of Good syndrome diagnosis until death or the last follow up. Survival curves were plotted using Kaplan-Meier method and compared with log-rank test. Cox proportional hazards model was further applied to test the significance of those prognostic factors regarding their survival impact.

To unravel homogeneous clinical subgroups within Good syndrome, a multiple correspondence analysis was first used to reduce the dimension of datasets. It cross-tabulated categorical variables and represented them graphically in a 2-dimensional Euclidean space by a multidimensional scaling technique (12). Input variables were chosen based on the defining criteria of Good syndrome, including thymoma, infection (type of pathogen and site of infection) and IgG level. After transformation of categorical variables into continuous variables (coordinates), we performed unsupervised hierarchical cluster analysis to determine clinical subgroups according to various characteristics. The clustering was constructed using Euclidean distance with the Ward agglomerative method. This method starts with each patient in its own cluster and the two most “similar” clusters based on Euclidean distance are combined at each step until the last two clusters are merged into a single cluster with all patients, as shown in a dendrogram (13, 14). Discriminative variables among clusters were further selected based on higher V test value with significant *p* value (15).

A decision tree was constructed with the use of chi-square automatic interaction detection technique to easily place the cases into different clusters (16). The *p* value was adjusted by Bonferroni correction. Ten-fold cross validation was applied to select the optimal tree. Performance of the decision tree was further evaluated by overall sensitivity and specificity of the cluster placement of cases.

All data were considered statistically significant at *p* < 0.05.

## Results

### General Characteristics

Our systematic literature review identified 162 patients from 121 records (17–137) (**Supplementary Figure 1**). The demographics, clinical features and outcomes are shown in **Table 1**. The median age of Good syndrome diagnosis was 58 years (IQR 51-67), and 51.0% were male. Because the ethnic origin of patient was not reported in most cases, geographic location of the corresponding author was recorded instead: Asia (n = 73, 45.1%), Europe (n = 58, 35.8%), North America (n = 22, 13.5%), Australia/Oceania (n = 5, 3.1%) and South America (n = 4, 2.5%). In 136 patients with available data regarding the chronological sequence of initial clinical presentation and subsequent Good syndrome diagnosis establishment, thymoma, infection and autoimmunity preceded the establishment of Good syndrome diagnosis in 44.9%, 29.4% and 25.7% patients, respectively. The median interval from initial clinical presentation to subsequent establishment of Good syndrome diagnosis was 24 months (IQR 4-72). Patients presented with infection (median interval 2 months, IQR 0-36) reached diagnosis earlier compared to those who presented with thymoma (median interval 36 months, IQR 6-82, *p* = 0.002) or autoimmunity (median interval 36 months, IQR 12-96, *p* = 0.001).

**Table 1:** Clinical characteristics, management, and outcomes of patients with Good syndrome.

Variables	N = 162
*Demographics*	
Age (median, IQR)	58 (51–67)
Male (%)	79 (51.0) (n = 155)
Country of cases (%)	
North America	22 (13.5)
South America	4 (2.5)
Asia	73 (45.1)
Europe	58 (35.8)
Australia/Oceania	5 (3.1)
*Index presentation*	*(n = 136)*
Thymoma (%)	61 (44.9)
Infections (%)	40 (29.4)
Autoimmunity (%)	35 (25.7)
*Index to diagnosis (median, IQR; months)*	*(n = 116)*
Overall	24 (4–72)
Thymoma	36 (6–82) (n = 52)
Infection	2 (0–36) (n = 31)
Autoimmunity	36 (12–96) (n = 33)
Thymoma classification* (%)	*(n = 121)*
WHO classification (%)	(n = 98)
Type A	23 (23.5)
Type B1/2/3	26 (26.5)
Type AB	49 (50.0)
Traditional classification (%)	(n = 6)
Spindle cell	2 (33.3)
Lymphocytic rich	1 (16.7)
Lymphoepithelial	2 (33.3)
Epithelial	1 (16.7)
Other classification (%)	(n = 17)
Benign	8 (47.1)
Malignant	9 (52.9)
*Thymoma staging (Masaoka system) (%)*	*(n = 22)*
Stage I	7 (31.8)
Stage II	8 (36.4)
Stage III	4 (18.2)
Stage IV	3 (13.6)
*Thymoma status when Good syndrome is diagnosed (%)*	*(n = 146)*
Disease free	75 (51.4)
Disease active	71 (48.6)
*Infections (%)*	*(n = 150 with infection)*
Classification	
Humoral defects	34 (22.7)
Cellular defects	86 (57.3)
Phagocytic defects	41 (27.3)
Others/unknown	72 (48.0)
Site	
Sinopulmonary tract	101 (67.3)
Eye	15 (10.0)
GI tract and liver	41 (27.3)
Urinary tract	11 (7.3)
Bone and joint	4 (2.7)
Skin and soft tissue	27 (18.0)
Central nerve system	12 (8.0)
Complicated bloodstream (with organ involvement)	13 (8.7)
Viremia without organ involvement	10 (6.7)
Mucosa**	21 (14.0)
*Autoimmunity (%)*	*(n = 83 with autoimmunity)*
Pure red cell aplasia	26 (31.3)
Myasthenia gravis	23 (27.7)
Lichen planus	19 (22.9)
*Diarrhea (%)*	*(n = 52 with diarrhea)*
Infection	30 (57.7)
Non-infection	22 (42.3)
*Second malignancy*	(n = 14 with concurrent second malignancy)***
Type	
Solid	6 (42.9)
Hematological	8 (57.1)
Timing of diagnosis	
Before Good syndrome	5 (35.7)
Concurrent with Good syndrome	3 (21.4)
After Good syndrome	6 (42.9)
*Laboratory findings (%)*	
Immunoglobulin (Ig)	
Low IgG	162 (100.0)
Low IgA	117 (86.0) (n = 136)
Low IgM	125 (92.6) (n = 135)
Complete blood counts	
Leukopenia	33 (44.0) (n = 75)
Anemia	45 (66.2) (n = 68)
Thrombocytopenia	6 (14.3) (n = 42)
Peripheral B cells	(n = 124)
Absent	55 (44.4)
Low	63 (50.8)
Normal	6 (4.8)
Peripheral T cells	
Low CD4	77 (71.3) (n = 108)
Elevated CD8	17 (20.5) (n = 83)
Inversed CD4/8 ratio	94 (82.5) (n = 114)
HIV test negativity	64 (100.0) (n = 64)
*Management (%)*	
Thymoma	(n = 150)
Thymectomy	135 (90.0)
Radiotherapy	10 (6.7)
Chemotherapy	12 (8.0)
Immunoglobulin replacement	127 (78.4)
Antimicrobial prophylaxis	28 (17.3)
Concurrent immunosuppressant	42 (50.6) (n = 83 with autoimmunity)
*Outcomes*	
Overall mortality	25 (15.4)
Infection	23 (92.0)
Others	2 (8.0)
10-year overall survival (%; 95% CI)	53.7 (25.8-75.1) (n = 109)

IQR, interquartile range; CI, confidence interval.

*Thymoma histological subtype was traditionally classified based on morphology alone. WHO classification was subsequently introduced, based on both the morphology of epithelial cells and the lymphocyte-to epithelial cell ratio.

**Mucosa included oral and vaginal mucosal infection, such as oral candidiasis.

***Fourteen pre-malignancy and malignancy were reported in 13 patients, including breast cancer (n = 1), nasopharyngeal cancer (n = 1), cutaneous Kaposi sarcoma (n = 1), lung cancer (squamous cell, n = 2; adenocarcinoma, n = 1), mucosa associated lymphoid tissue lymphoma (ocular adnexa, n = 1), monoclonal gammopathy of undetermined significance (n = 3), myelodysplastic syndrome (n = 3) and T cell large lymphocytic granular leukemia (n = 1).

### Clinical Features: Thymoma, Infection, Concurrent Autoimmunity and Second Malignancy

In 121 patients with thymoma histological classification data, WHO classification was used in 98 patients and type AB was found in 50.0%. Staging with the Masaoka system was only reported in 22 patients, and 68.2% were localized disease (stage I and II). At the time of Good syndrome diagnosis, half of the cases (51.4%) were in remission from thymoma standpoint.

Infections were recorded in 150 patients (92.6%), and the leading site was sinopulmonary tract (67.3%). Other common sites of infections included gastrointestinal system (27.3%), skin and soft tissue (18.0%), mucosa (14.0%) and eye (10.0%). Recovered pathogens are shown in **Supplementary Table 2**. The most frequently recorded bacterium, fungus and virus were *Pseudomonas* spp. (12.7%), *Candida* spp. (16.7%), and cytomegalovirus (24.7%), respectively. Given the diversity of pathogens reported, they were further categorized according to the major type of host defect predisposing to the infection (7–11) (**Supplementary Table 3**). As a result, 22.7%, 57.3% and 27.3% patients had infections related to humoral, cellular, and phagocytic defects.

Concurrent autoimmunity was identified in 83 patients (51.2%). Pure red cell aplasia (31.3%), myasthenia gravis (27.7%) and lichen planus (22.9%) were the most frequent disorders in these patients. Fourteen second pre-malignancy and malignancy were diagnosed in 13 patients (8.0%), before (n = 5), concurrent with (n = 3) and after (n = 6) diagnosis of Good syndrome. Eight were hematological and six were solid tumors.

### Laboratory Findings

All patients had hypogammaglobulinemia with a median IgG of 332 mg/dL (IQR 188-476) in those with reported levels (n = 126). Concurrent low IgA and IgM were noted in 86.0% (117/136) and 92.6% (125/135) patients with records. Absent or low peripheral B cells were shown in 118 of 124 patients (95.2%), as well as low absolute CD4 count in 77 of 108 patients (71.3%). Of 114 patients, 94 (82.5%) had an inverted CD4/8 ratio. The human immunodeficiency virus (HIV) was negative in all tested patients (n = 64).

There were no statistically significant differences in terms of immunoglobulin levels and peripheral B cell count between patients with and without infections related to humoral immunity defects. Regarding CD4 cell count and CD4/8 ratio, no significant differences were found between patients with and without infections related to cellular immunity defects (**Supplementary Table 4**).

### Overall Management

Of 150 patients with thymoma treatment data, 135 (90.0%) underwent thymectomy at the time of report. Chemotherapy and radiotherapy were given to 12 (8.0%) and 10 (6.7%) patients. In terms of immunoglobulin replacement, 127 patients (78.4%) received at least one dose. Antimicrobial prophylaxis was only used in 28 patients (17.3%). Of 83 patients with autoimmunity, 42 (50.6%) received concurrent immunosuppressive treatments (corticosteroid alone in 18 patients; immunosuppressants, such as cyclosporine, with or without corticosteroid in 24 patients).

### Outcomes and Prognostic Factors

A total of 25 patients (15.4%) died at the time of report. The main cause of mortality was infection (n = 23, 92.0%). In the whole cohort, factors impacting mortality consistently in both univariate and multivariate logistic regression included thymoma status (active disease, odds ratio [OR] 4.157, 95% confidence interval [CI] 1.219-14.177, *p* = 0.023), infection associated with cellular immunity defect (OR 3.324, 95% CI 1.100-10.046, *p* = 0.033), infection of sinopulmonary tract (OR 14.351, 95% CI 2.525-81.576, *p* = 0.003), infection of central nerve system (OR 6.403, 95% CI 1.205-34.027, *p* = 0.029) and infection of bloodstream (OR 6.917, 95% CI 1.519-31.505, *p* = 0.012) (**Table 2**).

**Table 2:** Univariate and multivariate analyses of factors impacting outcomes.

Variables	Logistic regression				Cox regression			
	Univariate		Multivariate		Univariate		Multivariate	
	OR (95% CI)	*P*	OR (95% CI)	*P*	HR (95% CI)	*P*	HR (95% CI)	*P*
Thymoma status: active disease	2.243 (0.886-5.667)	0.088	4.157 (1.219-14.177)	0.023	2.474 (1.000-6.119)	0.050	4.173 (1.584-10.990)	0.004
Infection: cellular immunity defects	2.609 (1.024-6.647)	0.044	3.324 (1.100-10.046)	0.033	3.172 (1.243-8.095)	0.016	2.895 (1.074-7.802)	0.036
Infection: sinopulmonary tract	5.384 (1.538-18.848)	0.008	14.351 (2.525-81.576)	0.003	3.439 (1.016-11.633)	0.047	4.312 (1.175-15.820)	0.028
Infection: central nerve system	4.643 (1.343-16.051)	0.015	6.403 (1.205-34.027)	0.029	4.051 (1.456-11.273)	0.007	3.424 (1.099-10.669)	0.034
Infection: bloodstream	4.031 (1.199-13.554)	0.024	6.917 (1.519-31.505)	0.012	3.707 (1.361-10.101)	0.010	3.832 (1.342-10.944)	0.012

OR, odds ratio; HR, hazard ratio; CI, confidence interval.

**Figure 1:**
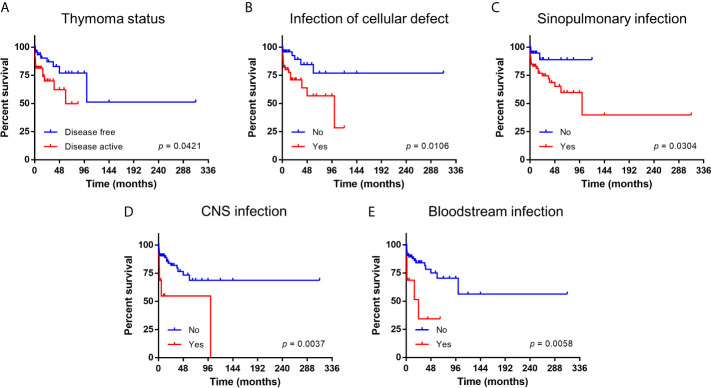
Kaplan-Meier survival curve of patients with Good syndrome stratified according to prognostic factors. **(A)** Thymoma status. **(B)** Infection related to cellular immunity defects. **(C)** Infection of sinopulmonary tract. **(D)** Infection of central nervous system (CNS). **(E)** Infection of bloodstream.

Of 109 patients with documented follow up duration (median 24 months, 95% CI 19-29 months), the 10-year overall survival was 53.7% (95% CI 25.8-75.1). The five prognostic factors identified by logistic regression in the whole cohort remained significant in these patients by both univariate and multivariate Cox regression (**Table 2** and **Supplementary Table 5**). Survival outcomes are depicted in **Figure 1**.

### Identification of Subgroups

**Figure 2:**
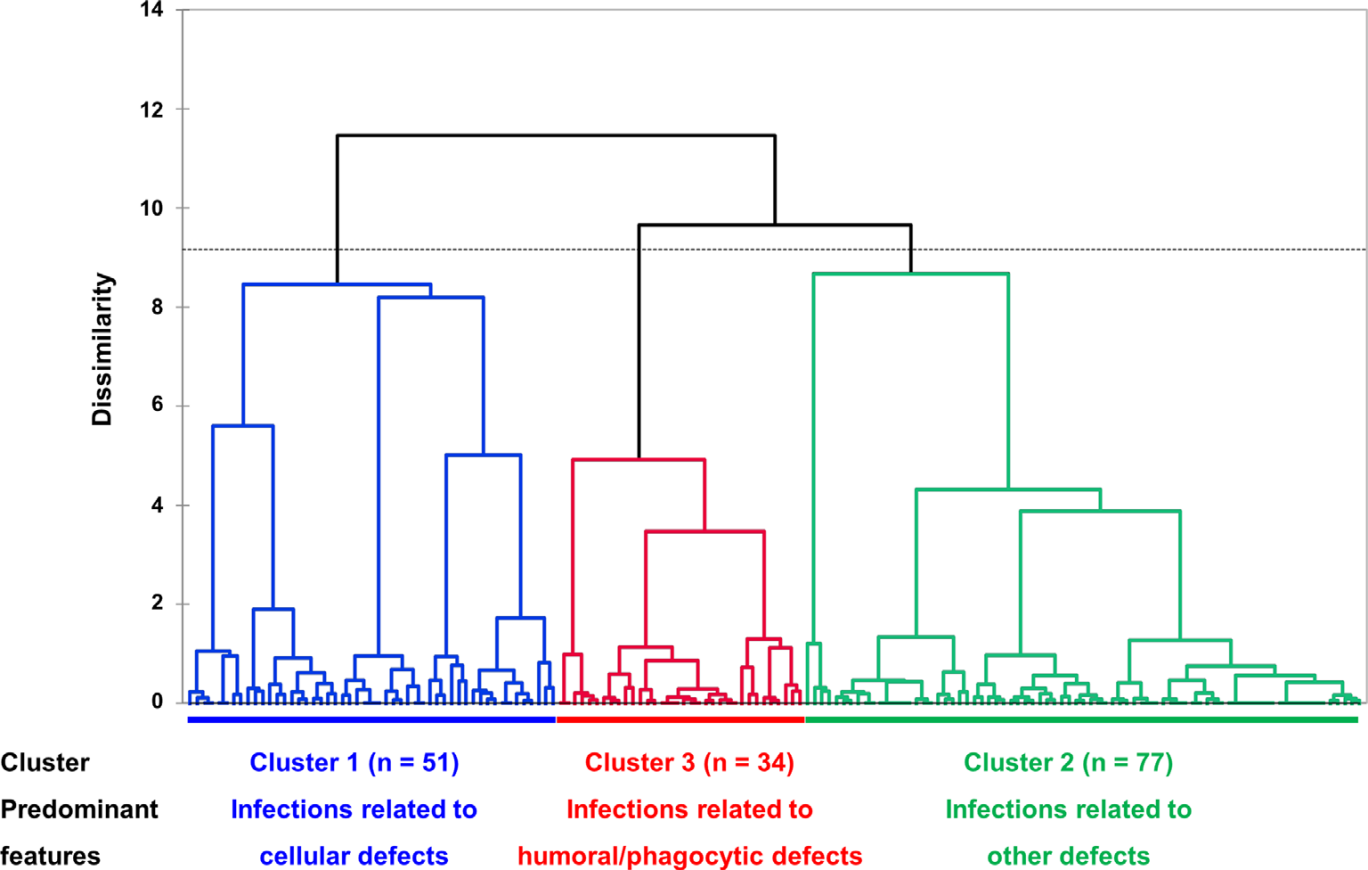
Dendrogram of unsupervised hierarchical clustering. Three clusters, based on similarity of cases, were identified, and represented in different colors (cluster 1, blue, infections related to cellular immunity defects; cluster 2, green, infections related to other immunity defects; cluster 3, red, infections related to humoral and phagocytic immunity defects). The vertical axis represents a measure of dissimilarity. In the horizontal axis, each patient is represented by a vertical line starting at the bottom and progressively merge with other patients to form clusters. Dashed line indicates the cut-off point for the three clusters.

Using multiple correspondence analysis and hierarchical clustering, we identified three clusters within the whole cohort (**Figure 2** and **Supplementary Figure 2**). Comparisons of clinical features among the three clusters are shown in **Table 3**. Cluster 1 (n = 51, 31.5%) was best characterized by more frequent infections related to cellular immunity defect (94.1%, *p* < 0.001; V test, 7.071), affecting mucosa (37.3%, *p* < 0.001; V test, 6.220) and central nerve system (23.5%, *p* < 0.001; V test, 5.295). The most discriminative variables of cluster 2 (n = 77, 47.5%) included infections related to other immunity defect (61.0%, *p* < 0.001; V test, 4.033) and more frequent IgG < 500 mg/dL (88.9%, *p* = 0.041; V test, 2.269). In terms of cluster 3 (n = 34, 21.0%), patients had more frequent infections related to humoral (67.7%, *p* < 0.001; V test, 7.493) and phagocytic defects (58.8%, *p* < 0.001; V test, 5.041), as well as involving sinopulmonary tract (91.2%, *p* < 0.001; 3.891). The outcomes were statistically different between patients of cluster 1 and 2 (OR 0.325, 95% CI 0.118-0.893, *p* = 0.029; hazard ratio 0.382, 95% CI 0.150-0.974, *p* = 0.044 for those who with follow up duration) (**Supplementary Figure 3**).

**Table 3:** Comparison of clinical characteristics among the three subgroups of patients with Good syndrome.

Variables (N, %)	Cluster 1 (n = 51)	Cluster 2 (n = 77)	Cluster 3 (n = 34)	P*
Age > 60 years	24 (47.1)	33 (42.9)	17 (50.0)	0.394
Male	25 (50.0) (n = 50)	35 (46.7) (n = 75)	19 (63.3) (n = 30)	0.300
Asian	21 (41.2)	38 (49.4)	14 (41.2)	0.580
Thymoma status: active	28 (58.3) (n = 48)	40 (56.3) (n = 71)	3 (11.1) (n = 27)	< 0.001
Infection: humoral defects	7 (13.7)	4 (5.2)	23 (67.6)	< 0.001
Infection: cellular defects	48 (94.1)	22 (28.6)	16 (47.1)	< 0.001
Infection: phagocytic defects	11 (21.6)	10 (13.0)	20 (58.8)	< 0.001
Infection: others	17 (33.3)	47 (61.0)	8 (23.5)	< 0.001
Infection: sinopulmonary	24 (47.1)	46 (59.7)	31 (91.2)	< 0.001
Infection: eye	12 (23.5)	0 (0.0)	3 (8.8)	< 0.001
Infection: GI and liver	11 (21.6)	20 (26.0)	10 (29.4)	0.705
Infection: urinary tract	8 (15.7)	2 (2.6)	1 (2.9)	0.010
Infection: bone and joint	0 (0.0)	4 (5.2)	0 (0.0)	0.104
Infection: skin and soft tissue	10 (19.6)	16 (20.8)	1 (2.9)	0.053
Infection: central nerve system	12 (23.5)	0 (0.0)	0 (0.0)	< 0.001
Infection: bloodstream	5 (9.8)	0 (0.0)	8 (23.5)	< 0.001
Infection: viremia	10 (19.6)	0 (0.0)	0 (0.0)	< 0.001
Infection: mucosa	19 (37.3)	0 (0.0)	2 (5.9)	< 0.001
Autoimmunity: lichen planus	5 (9.8)	13 (16.9)	1 (2.9)	0.096
Autoimmunity: MG	8 (15.7)	9 (11.7)	6 (17.6)	0.663
Autoimmunity: PRCA	9 (17.6)	10 (13.0)	7 (20.6)	0.562
Autoimmunity: others	6 (11.8)	14 (18.2)	1 (2.9)	0.084
IgG < 500 mg/dL	29 (69.0) (n = 42)	56 (88.9) (n = 63)	20 (80.0) (n = 25)	0.041
Low IgA	34 (77.3) (n = 44)	57 (89.1) (n = 64)	26 (92.9) (n = 28)	0.112
Low IgM	39 (90.7) (n = 43)	61 (96.8) (n = 63)	25 (86.2) (n = 29)	0.166
Absent peripheral B cells	18 (47.4) (n = 38)	22 (38.6) (n = 57)	15 (51.7) (n = 29)	0.462
Low CD4	26 (70.3) (n = 37)	30 (63.8) (n = 47)	21 (87.5) (n = 24)	0.112
Inverted CD4/CD8 ratio	31 (83.8) (n = 37)	39 (78.0) (n = 50)	24 (88.9) (n = 27)	0.471
Thymectomy	43 (86.0) (n = 50)	65 (90.3) (n = 72)	27 (96.4) (n = 28)	0.336
Radio- and/or chemotherapy	11 (22.0) (n = 50)	8 (11.1) (n = 72)	3 (10.7) (n = 28)	0.199
Immunoglobulin replacement	33 (64.7)	62 (80.5)	32 (94.1)	0.004
Antimicrobial prophylaxis	10 (19.6)	10 (13.0)	8 (23.5)	0.347
Immunosuppressant (n = 83)**	15 (60.0) (n = 25)	19 (43.2) (n = 44)	8 (57.1) (n = 14)	0.351

GI, gastrointestinal; MG, myasthenia gravis; PRCA, pure red cell aplasia; Ig, immunoglobulin.

*Global p value.

**In patients with concurrent autoimmune disorders.

**Figure 3:**
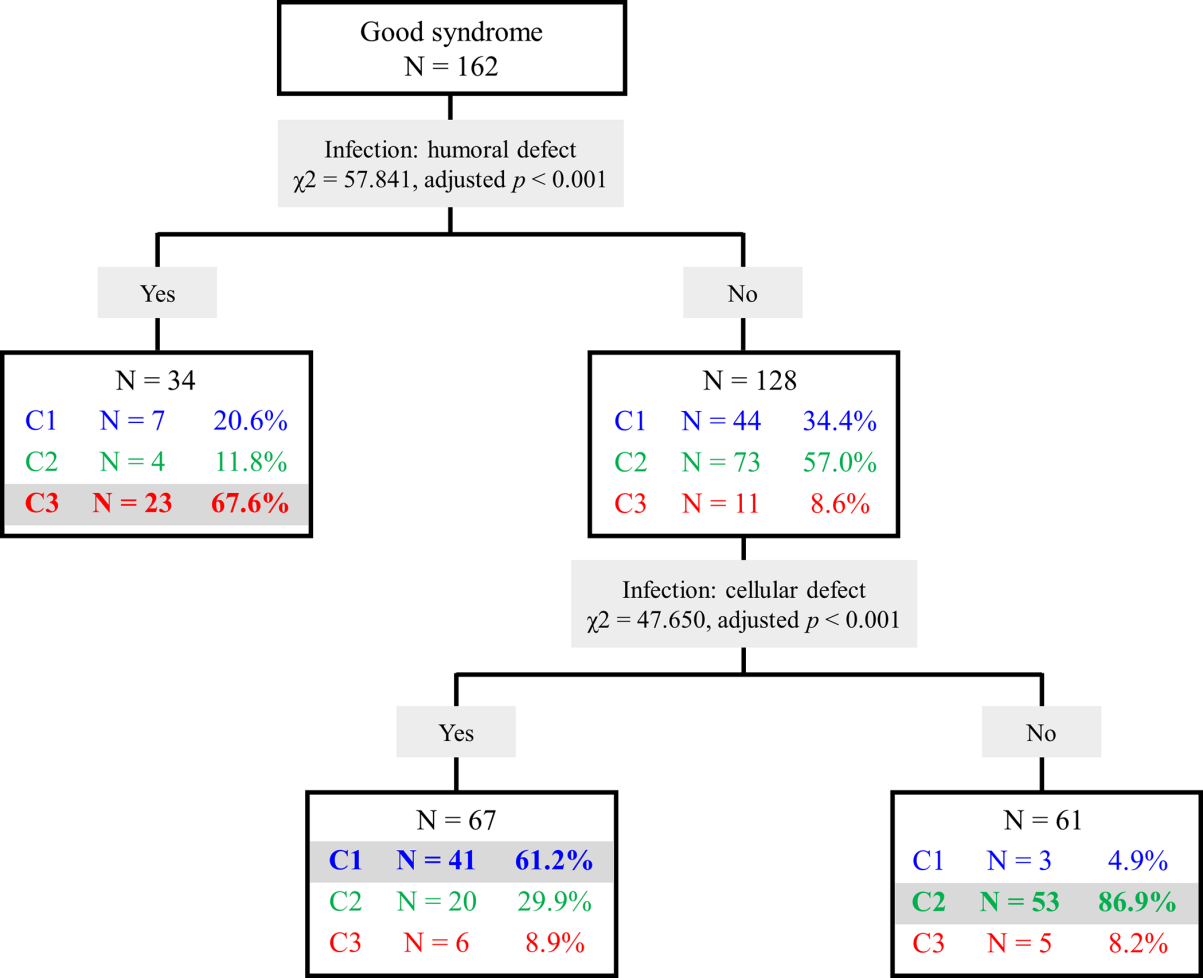
Decision tree of Good syndrome classification. Cluster 1 (C1), infections related to cellular immunity defects; Cluster 2 (C2), infections related to other immunity defects; Cluster 3 (C3), infections related to humoral and phagocytic immunity defects.

To easily classify cases into the three clusters, a decision tree was created by screening discriminative variables among the three clusters (**Figure 3**). Using infections related to humoral (χ2 = 57.841, adjusted *p* < 0.001) and cellular defects (χ2 = 47.650, adjusted *p* < 0.001), the tree showed 72.2% correct estimation with an overall sensitivity of 72.3% and specificity of 86.2%.

## Discussion

The current systematic review summarized clinical data of a recent and large series of patients with Good syndrome. To the best of our knowledge, this is the first study to comprehensively explore the independent prognostic factors and define clinical subgroups within this rare adult-onset immunodeficiency. We found thymoma status and infection type as well as site impacting the outcome. Distributions of these features varied significantly among subgroups identified *via* hierarchical clustering, supporting the relevance to define these subgroups in clinical practice. Otherwise, the findings of current study were comparable to those of a previous systematic review including cases published between 1956 and 2009 (2) as well as four case series reported in the recent decade (3–6) (**Supplementary Table 1**), in terms of demographics, thymoma classification, infection sites and pathogens, concurrent autoimmunity and laboratory findings of immunodeficiency, reinforcing our understanding of this rare disorder.

Notably, we introduced thymoma status and infection classification in our study, both turned to be critical in the subsequent prognostic and clustering analyses. First, although the exact role of thymoma in Good syndrome development remains unclear, it likely disrupts the balance between host-defense and self-tolerance. Given the crucial physiological role of thymus in T cell education, concurrent autoimmunity and immunodeficiency observed in the context of thymoma reflects both over-reactivity to self-antigens and hypo-reactivity to pathogens (138). Patients with thymoma requiring treatment (e.g., thymectomy, chemotherapy and/or radiotherapy) at Good syndrome diagnosis showed inferior outcomes, which may suggest a different underlying immunological status but could also be attributed to more complicated clinical needs, especially in the context of active infections. It is noteworthy that hypogammaglobulinemia did not improve in all patients of the current series with IgG level measured after thymectomy (n = 92), indicating thymoma management alone is not sufficient to resolve this disorder.

Second, we classified infection into different subtypes according to the pathogens, given its huge diversity and limited cases of each pathogen making statistical analysis less feasible in this rare disease. The classification is based on our current knowledge of predisposing immunological factors related to infection of each pathogen, considering the typical infections observed in patients with X-linked agammaglobulinemia (humoral defect) (7), acquired immunodeficiency syndromes (AIDS, cellular defect) (8) and neutropenia (such as chemotherapy induced) (9) as well as neutrophil dysfunction in chronic granulomatous disease (phagocytic defect) (10) as prototypes. Of note, infection related to cellular immunity defects was the predominant type, emphasizing Good syndrome is a combined immunodeficiency. Although low CD4 cell count and inverted CD4/8 ratio were commonly found in these patients, both were not prognostic and their association with the onset of opportunistic infection was not established. Indeed, these infections occurred even in those who had preserved CD4 cell count, as noted in a recent French Good syndrome series (3). In addition, all patients had hypogammaglobulinemia, but the level of IgG was neither prognostic nor differed in terms of infections related to humoral defects. Even in patients with common variable immunodeficiency, the prognostic value of baseline IgG remains controversial (139, 140). Overall, the type of infections discriminates the three clusters defined in our study and could be used to easily place a patient into the corresponding cluster (cluster 1, infections related to cellular immunity defects; cluster 2, infections related to other immunity defects; cluster 3, infections related to humoral and phagocytic immunity defects). The enrichment of clinical features suggests a shared underlying pathogenesis in each cluster, which was not well reflected with the use of routine immunological workups, including serum immunoglobulin levels and lymphocyte subset enumeration of peripheral blood. These observations indicate the requirement of further workups to particularly assess functions of different immune cell subsets. Unfortunately, more advanced immunological workups to evaluate B (e.g., vaccination response), T (e.g., lymphocyte proliferation to mitogens) and phagocytic cell (e.g., respiratory burst) functions were not available in most cases. Moreover, anti-cytokine autoantibodies seem to be crucial links between thymoma and cellular immunity defects, predisposing to various opportunistic infections (141). Their roles in Good syndrome pathogenesis and disease evolution need further studies. In addition, whether there is underlying molecular defect predisposing to Good syndrome also remains underexplored.

Although infection remained the leading cause of death, overall mortality of Good syndrome showed significant improvement in the current series (15.4%) compared to the previous one (46.1%) (2), which could be multifactorial and related to improved recognition of this rare disorder, more frequent use of immunoglobulin replacement, expanded availability of various antimicrobial agents as well as better supportive cares in the recent decade. Majority of patients underwent thymectomy, which unfortunately did not resolve the hypogammaglobulinemia. Immunoglobulin replacement was used in 78.4% patients for at least one dose, although it did not impact prognosis in the current series. Given the description in most case reports, it is hard to know whether these patients received a regular long-term replacement. Moreover, infection related to cellular immunity defects, the main prognostic factor, is not affected by immunoglobulin replacement. Antimicrobial prophylaxis was less commonly used in this series, which deserves further studies to address its potential significance, given the efficacy and safety of low dose antibiotics prophylaxis have been demonstrated in patients with primary antibody deficiencies (142).

Our study has limitations given its design. Although the current series included the largest number of cases to date, analyses were restricted to what was reported in the literature (i.e., unavoidable reporting bias and missing data), and limited events as well as incomplete follow up may also underpower our analyses. Therefore, the results should not be over-interpreted. Independent prognostic factors as well as the subgroup clustering requires external validations. More investigational studies are needed to unravel the unique pathogenesis of each cluster, such as functions of different immune cell subsets and the presence of a specific set of anti-cytokine antibodies.

In conclusion, Good syndrome is a rare combined immunodeficiency in adults that has subgroups with different clinical features. Infection type and site impact its overall survival. Future studies should explore whether each subgroup has unique disease pathogenesis that may be amenable to more specific treatments.

## Data Availability Statement

The original contributions presented in the study are included in the article/**Supplementary Material**. Further inquiries can be directed to the corresponding author.

## Author Contributions

YS conceived the study, collected the records, analyzed the data, and contributed to the manuscript. CW conceived the study, collected the records, analyzed the data, and wrote the manuscript. All authors contributed to the article and approved the submitted version.

## Conflict of Interest

The authors declare that the research was conducted in the absence of any commercial or financial relationships that could be construed as a potential conflict of interest.
