# Supplementary material for: When the Good Syndrome Goes Bad: A Systematic Literature Review
Source: Front Immunol. 2021 May 25;12:679556. doi: 10.3389/fimmu.2021.679556 (PMC8185358; doi:10.3389/fimmu.2021.679556)

**Supplementary table 1**. Good syndrome series with aggregated data reported between 2010-2020

|  | **DEFicit Immunitaire de l’adulte (DFEI)** | | **Good syndrome study group** | | **Chinese series** | | **UK-Primary Immune Deficiency (UKPID)** |
| --- | --- | --- | --- | --- | --- | --- | --- |
| ***Cohort information*** |  | |  | |  | |  |
| Study design | French national prospective multicenter registry | | Multinational multicenter questionnaire-based survey | | Literature review limited to studies from China | | UK national prospective multicenter registry |
| Study period | April 2004 - June 2013 | | 2012 and 2014 | | Up to January 2017 | | 2009 - 2018 |
| Enrollment criteria | Thymoma plus an antibody production defect (any class or subclass of immunoglobulin) | | Thymoma plus hypogammaglobulinemia | | Thymoma plus adult-onset immunodeficiency (hypogammaglobulinemia, low-absent B cells and variable defects in cellular immunity) | | Thymoma plus hypogammaglobulinemia |
| Number of patients | 21 | | 47 | | 47 | | 78 |
| ***Demographics*** |  | |  | |  | |  |
| Age (years, median) |  | |  | |  | |  |
| First symptom | 56 | | NA | | NA | | 54 |
| Thymoma | 57 | | 58 | | NA | | 58 |
| Hypogammaglobulinemia | 60 | | NA | | NA | | 58 |
| Evaluation/Diagnosis | 64 | | 58 | | 55 | | 64 |
| Male (%) | 12 (57.1) | | 23 (48.9) | | 16 (34.0) | | 32 (41.0) |
| ***Infections*** |  | |  | |  | |  |
| All (%) | 20 (95.2) | | 46 (97.9) | | 42 (89.4) | | NA |
| Most typical site: sinopulmonary (%) | 18 (85.7) | | 35 (74.5) | | 31 (66.0) | | 74 (94.9) |
| Most typical pathogens (% of cases with infections) | |  | |  | |  | |
| Bacteria: S. pneumonia | 7 (35.0) | | 12 (26.1) | | NA | | NA |
| Virus: CMV | 4 (20.0) | | 6 (13.0) | | 7 (16.7) | | 3 (NA) |
| Fungus: Candida | 5 (25.0) | | 9 (19.6) | | NA | | 3 (NA) |
| ***Immunodeficiency*** |  | |  | |  | |  |
| Immunoglobulin level (g/L, median) | |  | |  | |  | |
| IgG | 3.35 | | 2.28 | | NA | | 2.92 |
| IgA | 0.14 | | 0.25 | | NA | | 0.2 |
| IgM | 0.17 | | 0.1 | | NA | | 0.09 |
| Low-absent peripheral B-cell (%) | 20 (95.2) | | 36 (94.7; n = 38) | | 42 (100; n = 42) | | 78 (100) |
| CD4 (x10^6/L, median) | 404 | | 565 | | NA* | | 530 |
| ***Thymoma*** |  | |  | |  | |  |
| WHO classification: A/B/AB/C (%) | NA | | 14 (37.8)/9 (24.3)/13 (35.1)/1 (2.7) (n = 37) | | 10 (35.7)/3 (10.7)/14 (50.0)/1 (3.6) (n = 28) | | 4 (8.7)/6 (13.0)/27 (58.7)/9 (19.6) (n = 46) |
| ***Autoimmunity*** |  | |  | |  | |  |
| All (%) | 16 (76.2) | | 24 (51.1) | | 17 (36.2) | | 20 (25.6) |
| Most common concurrent autoimmune manifestation: PRCA (%) | 7 (33.3) | | 8 (17.0) | | 8 (17.0) | | 10 (12.8) |
| ***Chronic diarrhea (including both infectious and non-infectious causes)*** | 8 (38.1) | | NA | | 17 (36.2) | | 2 (2.6) |
| ***Concurrent malignancy*** |  | |  | |  | |  |
| Lymphoma (%) | 0 (0) | | 1 (2.1; T-LGL) | | NA | | 0 (0) |
| Others (%) | 1 (4.7; brain) | | 4 (8.5)** | | NA | | 6 (7.7)*** |
| ***Treatment*** |  | |  | |  | |  |
| Thymectomy (%) | 19 (90.5) | | 37 (78.7) | | 36 (76.6) | | 78 (100) |
| Immunoglobulin replacement (%) | 13 (61.9) | | 44 (93.6) | | NA | | 78 (100) |
| Antimicrobial prophylaxis (%) | NA | | 29 (61.7) | | NA | | NA |
| ***Outcomes*** |  | |  | |  | |  |
| Death (%) | 6 (28.6) | | 16 (41.0; n = 39) | | NA | | 7 (9.0) |
| 10-year survival (%) | 84 | | 68 | | NA | | NA |

Abbreviations: NA, not available; CMV, cytomegalovirus; Ig, immunoglobulin; PRCA, pure red cell aplasia; T-LGL, T-cell large granular lymphocyte leukemia.

Data are presented as number (%) unless otherwise specified.

* 37/39 (94.9) had low CD4 cell counts.

** Skin (n = 2), thyroid (n = 1), unknown (n = 1).

*** Skin (n = 2), kidney (n = 1), breast (n = 1), cervix (n = 1), lung (n = 1).

**Supplementary table 2**. List of pathogens recovered from patients with Good syndrome

| **Pathogens** | **N (%) (n = 150 with infections)** |
| --- | --- |
| Bacterium |  |
| *Pseudomonas spp.* | 19 (12.7) |
| *Hemophilus influenza* | 14 (9.3) |
| *Streptococcus pneumoniae* | 13 (8.7) |
| *Campylobacter spp.* | 10 (6.7) |
| *Mycobacterium tuberculosis* | 10 (6.7) |
| *Staphylococcus aureus* | 9 (6.0) |
| *Escherichia coli* | 8 (5.3) |
| *Klebsiella spp.* | 4 (2.7) |
| *Acinetobacter baumannii* | 3 (2.0) |
| *Clostridium difficile* | 3 (2.0) |
| *Enterobacter spp.* | 2 (1.3) |
| *Bordetella spp.* | 2 (1.3) |
| *Nocardia spp.* | 2 (1.3) |
| *Non-tuberculous mycobacteria* | 2 (1.3) |
| Group A streptococcus | 1 (0.7) |
| Group B streptococcus | 1 (0.7) |
| *Legionella pneumophila* | 1 (0.7) |
| *Moraxella spp.* | 1 (0.7) |
| *Mycoplasma hominis* | 1 (0.7) |
| *Proteus spp.* | 1 (0.7) |
| *Staphylococcus epidermis* | 1 (0.7) |
| *Streptococcus oralis* | 1 (0.7) |
| *Salmonella spp.* | 1 (0.7) |
| Fungus |  |
| *Candida spp.* | 25 (16.7) |
| *Pneumocystis jiroveci* | 12 (8.0) |
| *Aspergillus spp.* | 8 (5.3) |
| *Cryptococcus spp.* | 2 (1.3) |
| *Histoplasma spp.* | 1 (0.7) |
| *Mucor spp.* | 1 (0.7) |
| Virus |  |
| *Cytomegalovirus* | 37 (24.7) |
| *Herpes simplex virus* | 10 (6.7) |
| *Varicella zoster virus* | 4 (2.7) |
| *John Cunningham virus* | 3 (2.0) |
| *Epstein-Barr virus* | 2 (1.3) |
| *Hepatitis B virus* | 2 (1.3) |
| *Norovirus* | 1 (0.7) |
| *Rhinovirus* | 1 (0.7) |
| Parasite |  |
| *Giardia spp.* | 3 (2.0) |
| *Isospora belli* | 2 (1.3) |
| *Leishmania spp* | 2 (1.3) |
| *Toxoplasma spp.* | 2 (1.3) |

**Supplementary table 3**. Pathogen classifications based on underlying immune defects

| **Classifications** | **Pathogens** |
| --- | --- |
| Infection: humoral immunity defects* | *Streptococcus pneumonia, Hemophilus influenza, Streptococcus oralis, Campylobacter spp., Moraxella spp., Bordetella spp., Mycoplasma hominis, Group A streptococcus.*  *Norovirus, Rhinovirus.*  *Giardia spp.* |
| Infection: cellular immunity defects** | *Mycobacteria (TB/NTM), Salmonella spp., Legionella pneumophila.*  *Candida spp., Pneumocystis jiroveci, Cryptococcus spp., Histoplasma spp.*  *Cytomegalovirus, Epstein-Barr virus, Herpes simplex virus, John Cunningham virus, Varicella zoster virus.*  *Isospora belli, Leishmania spp., Toxoplasma spp.* |
| Infection: phagocytic defects*** | *Pseudomonas spp., Escherichia coli, Klebsiella spp., Enterobacter spp., Proteus spp, Nocardia spp. Staphylococcus aureus.*  *Aspergillus spp., Mucor spp.* |
| Infection: others or unknown pathogens | *Acinetobacter baumannii, Clostridium difficile, Staphylococcus epidermis, Group B streptococcus.*  *Hepatitis B virus.*  Unknown pathogens. |

* Infections that predominately requiring antibody-mediated immunity in clearance (i.e., more commonly seen in patients with humoral immunity defect, such as X-linked agammaglobulinemia).

** Infections that predominately requiring cell-mediated immunity in clearance (i.e., more commonly seen in patients with cellular immunity defect, such as acquired immunodeficiency syndrome).

*** Infections that predominately requiring phagocytic response in clearance (i.e., more commonly seen in patients with neutropenia and/or phagocyte dysfunction, such as chemotherapy related neutropenia and chronic granulomatous disease).

**Supplementary table 4**. Immunological findings in patients with different types of infection

| **Infection: cellular immunity defects** | **Yes (n = 86)** | **No (n = 76)** | ***P*** |
| --- | --- | --- | --- |
| Low CD4 cells | 47 (n = 64) | 30 (n = 44) | 0.553 |
| Inverted CD4/CD8 ratio | 52 (n = 62) | 42 (n = 52) | 0.665 |
| **Infection: humoral immunity defects** | **Yes (n = 34)** | **No (n = 128)** | ***P*** |
| IgG level (median, IQR) (mg/dL) | 337 (171-418) (n = 26) | 327 (189-480) (n = 100) | 0.435 |
| Low IgA | 26 (n = 30) | 91 (n = 106) | 0.909 |
| Low IgM | 27 (n = 30) | 98 (n = 105) | 0.539 |
| Absent peripheral B cells | 16 (n = 29) | 39 (n = 95) | 0.180 |
| Absent-low peripheral B cells | 28 (n = 29) | 90 (n = 95) | 0.690 |
| **Infection: phagocytic defects** | **Yes (n = 41)** | **No (n = 121)** | ***P*** |
| Leukopenia | 14 (n = 23) | 19 (n = 52) | 0.088 |

Abbreviations: IQR, interquartile range; Ig, immunoglobulin.

**Supplementary table 5**. Univariate analysis of logistic regression for factors impacting survival

| **Variable** | **Odds ratio** | **95% confidence interval** | ***P*** |
| --- | --- | --- | --- |
| Years of publications (2016-2020) | 1.369 | 0.581-3.229 | 0.473 |
| Age (continuous) | 1.021 | 0.985-1.059 | 0.258 |
| Male | 0.784 | 0.327-1.877 | 0.585 |
| Area: Asia | 0.950 | 0.403-2.242 | 0.908 |
| Index presentation: infection | 1 |  |  |
| thymoma | 1.155 | 0.412-3.238 | 0.785 |
| autoimmunity | 0.975 | 0.294-3.236 | 0.967 |
| Index presentation to diagnosis > 2 years | 1.719 | 0.637-4.641 | 0.285 |
| Thymoma status: active disease | 2.243 | 0.886-5.677 | 0.088 |
| Infection: humoral immunity defects | 0.931 | 0.322-2.693 | 0.895 |
| Infection: cellular immunity defects | 2.609 | 1.024-6.647 | 0.044 |
| Infection: phagocytic defects | 3.438 | 1.418-8.331 | 0.006 |
| Infection: others/unknown | 1.432 | 0.609-3.366 | 0.410 |
| Infection: sinopulmonary | 5.384 | 1.538-18.848 | 0.008 |
| Infection: eye | 0.829 | 0.175-3.923 | 0.814 |
| Infection: GI and liver | 0.701 | 0.245-2.007 | 0.508 |
| Infection: skin and soft tissue | 0.642 | 0.178-2.319 | 0.499 |
| Infection: central nerve system | 4.643 | 1.343-16.051 | 0.015 |
| Infection: bloodstream | 4.031 | 1.199-13.554 | 0.024 |
| Infection: viremia | 0.593 | 0.072-4.895 | 0.627 |
| Infection: mucosa* | 3.417 | 1.216-9.602 | 0.020 |
| Diarrhea: no | 1 |  |  |
| yes (infection) | 0.500 | 0.138-1.812 | 0.291 |
| yes (non-infection) | 0.450 | 0.097-2.083 | 0.307 |
| Autoimmunity: lichen planus | 1.549 | 0.468-5.124 | 0.473 |
| Autoimmunity: myasthenia gravis | 0.480 | 0.105-2.191 | 0.344 |
| Autoimmunity: pure red cell aplasia | 2.415 | 0.890-6.555 | 0.083 |
| Second pre-malignancy and malignancy | 1.732 | 0.441-6.797 | 0.431 |
| IgG (continuous) | 0.999 | 0.996-1.002 | 0.572 |
| Normal-elevated IgA | 1.103 | 0.290-4.195 | 0.886 |
| Normal-elevated IgM | 0.620 | 0.074-5.179 | 0.659 |
| Presence of peripheral B cells | 0.577 | 0.200-1.664 | 0.309 |
| Normal CD4 | 0.802 | 0.238-2.711 | 0.723 |
| Normal CD4/8 ratio | 1.008 | 0.261-3.899 | 0.990 |
| Thymectomy* | 0.308 | 0.094-1.004 | 0.051 |
| Radio- and/or chemotherapy | 1.287 | 0.392-4.220 | 0.678 |
| Immunoglobulin replacement | 1.121 | 0.388-3.238 | 0.832 |
| Antimicrobial prophylaxis | 0.611 | 0.170-2.201 | 0.451 |
| Concurrent immunosuppressants | 1.133 | 0.437-2.941 | 0.797 |

* Statistically significant correlations: infection of mucosa and infection related to cellular immunity defect (*p* < 0.001); thymoma status and thymectomy (*p* < 0.001).

**Supplementary Figure 1**. PRISMA flow diagram of record identification, screening and inclusion.


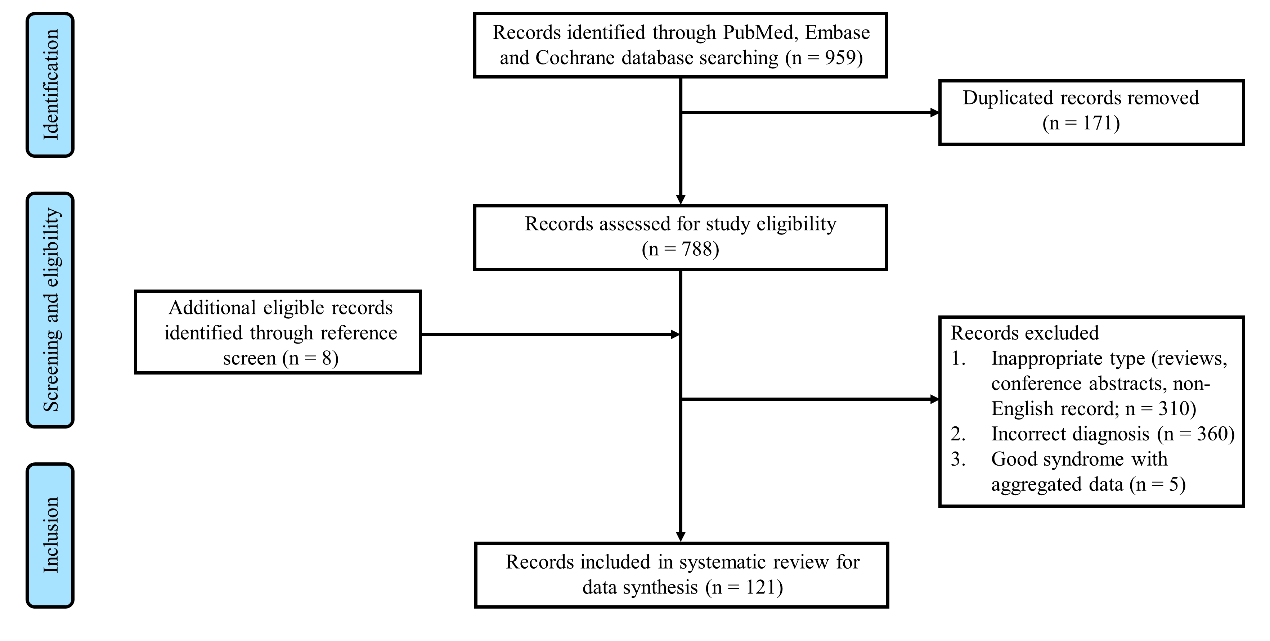


**Supplementary Figure 2**. Factor maps of individual patient represented on two dimensions of multiple correspondence analysis.


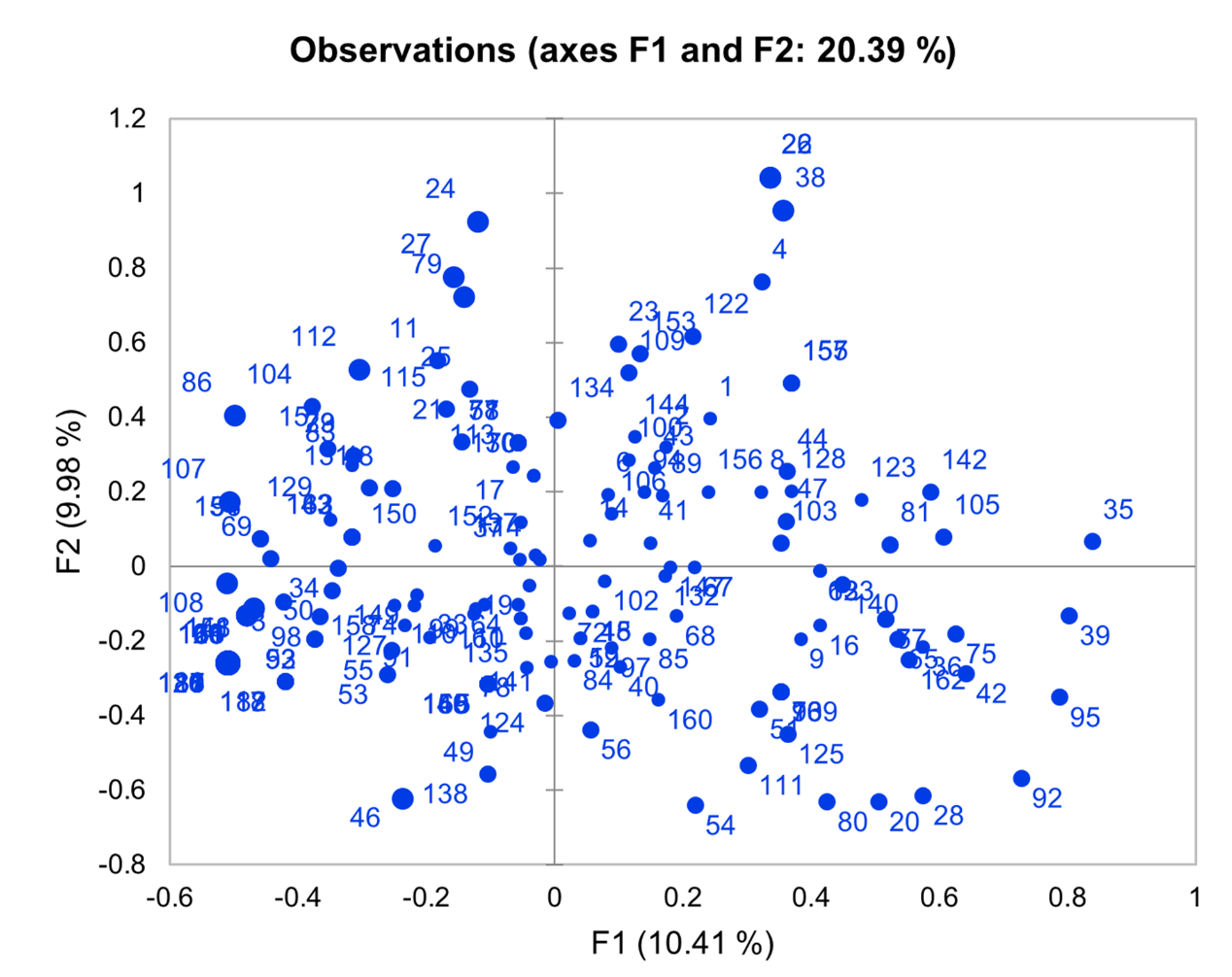


**Supplementary Figure 3**. Kaplan-Meier survival curve of patients in each cluster. Cluster 1, infections related to cellular immunity defects; Cluster 2, infections related to other immunity defects; Cluster 3, infections related to humoral and phagocytic immunity defects.


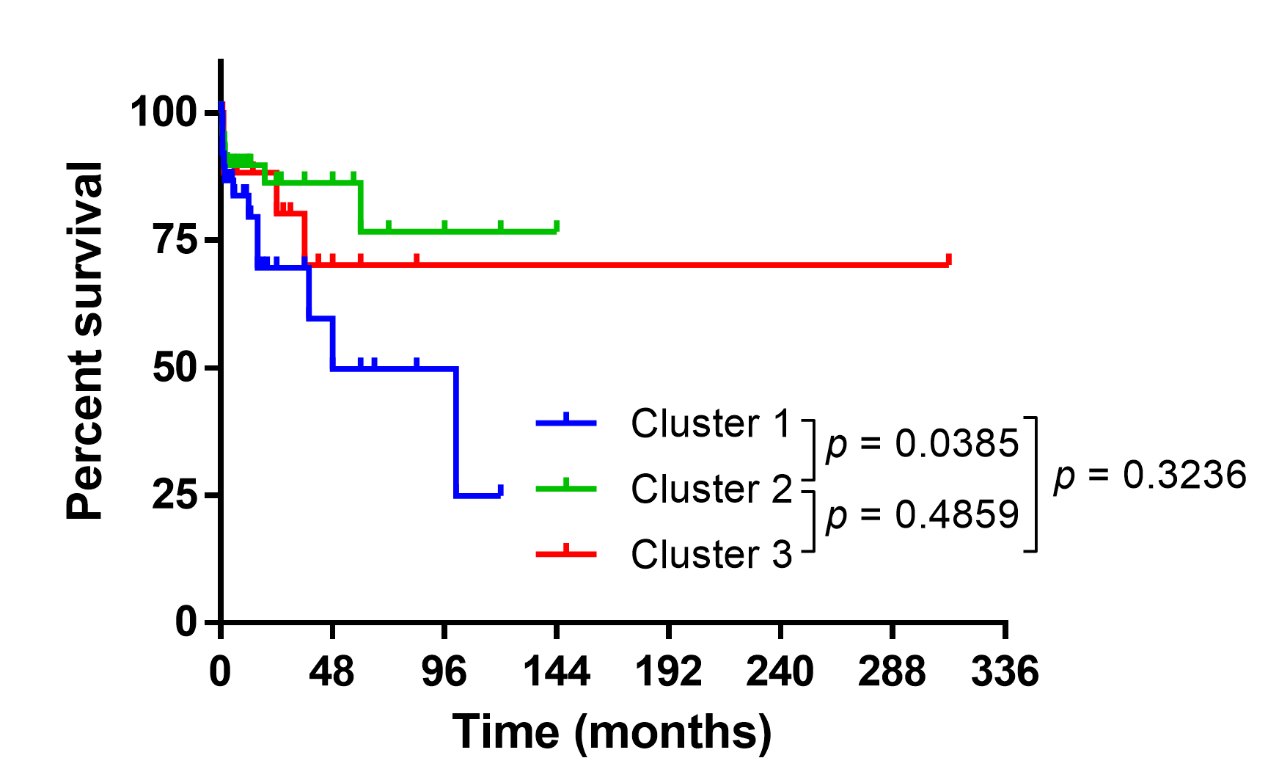

Supplement: Supplementary file 1 [file DataSheet_1.docx]
